# The Oval‐like Cross‐section of Femoral Neck Isthmus in Three‐dimensional Morphological Analysis

**DOI:** 10.1111/os.12914

**Published:** 2021-01-08

**Authors:** Ru‐yi Zhang, Yan‐peng Zhao, Xiu‐yun Su, Jian‐tao Li, Jing‐xin Zhao, Li‐cheng Zhang, Pei‐fu Tang

**Affiliations:** ^1^ Department of Orthopaedics Shijingshan Teaching Hospital of Capital Medical University, Beijing Shijingshan Hospital Beijing China; ^2^ Medical School of Chinese PLA Beijing China; ^3^ Department of Orthopaedics Chinese PLA General Hospital, National Clinical Research Center for Orthopedics, Sports Medicine & Rehabilitation Beijing China; ^4^ Department of Orthopaedics Southern University of Science and Technology Hospital Shenzhen China

**Keywords:** 3D, Area, Cross‐section, Femoral neck isthmus, Oval‐like

## Abstract

**Objectives:**

To investigate the cross‐section shape of the femoral neck isthmus (FNI) in three‐dimensional reconstruction model of the femoral neck.

**Methods:**

From December 2009 to December 2012, computed tomography (CT) data of bilateral hip joint from 200 consecutive patients (137 males and 63 females, 69.41 ± 9.21 years old, ranged from 50–85 years old) who underwent surgical treatments for proximal femoral fracture were retrospectively reviewed. The 3D model of the proximal femur was reconstructed, and the “inertia axis” method, which was applied to measure the long and short axes of the cross‐section of the FNI, was established. The cross‐sectional area and perimeter were calculated by a formula using the length of the long and short axes and then compared with the actual measured values by the software. Correlation between the descriptive parameters of the FNI cross‐section (area, perimeter, and eccentricity) and patients' demographics (age, height, and weight) was analyzed. Stepwise linear regression analysis was used to determine the main relevant factors.

**Results:**

The ICC results showed excellent data reproducibility ranged from 0.989 to 0.996. There was no significant difference in the cross‐sectional area of the FNI between the actual measured values and the predicted values using the formula (732.83 ± 126.74 mm^2^ *vs* 731.62 ± 128.15 mm^2^, *P* = 0.322). The perimeter using the two methods showed narrow while significant difference (97.86 ± 8.60 mm *vs* 92.84 ± 8.65 mm, *P* < 0.001), the actual measured values were about 5 mm greater than the predicted values. The parameters (area, perimeter, and eccentricity) were significantly larger in male than female (*P* < 0.001). A positive correlation between the cross‐sectional area, perimeter, height, and weight was observed. The stepwise linear regression analysis showed that the regression equation of the FNI area was as follows: *Y* = −1083.75 + 1033.86 × HEIGHT + 1.92 × WEIGHT, *R* ^2^ = 0.489.

**Conclusion:**

The cross‐section shape of the FNI appears to be oval‐like in the 3D model, which is separated according to the inertia axis, and the findings proposed an anatomical basis for the further study of the spatial configuration of cannulated screws in the treatment of femoral neck fractures.

## Graphical abstract

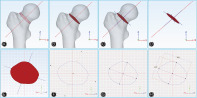

The morphological features of the cross‐section of the femoral neck, especially the femoral neck isthmus (FNI), are of great significance for the spatial configuration of the screws used in the internal fixation of femoral neck fractures. This study utilizes the three‐dimensional model of the proximal femur to segment the FNI plane. We found that the actual measured data of FNI cross‐section was in line with the values calculated using elliptical equations, thus proving that FNI cross‐section was approximately elliptical.

## Introduction

Femoral neck fracture is a common fracture in orthopaedic trauma, which occurred in approximately 3.61% of the systemic fracture and 50%–60% of the hip fracture^1^. For young and middle‐aged patients of femoral neck fractures without severe fracture displacement, internal fixation is the preferred choice in current surgical strategies^2^, ^3^, ^4^. Internal fixation with cannulated screws parallel to the femoral neck axis has been a well‐accepted approach as the surgical treatment for femoral neck fractures in clinical practices^5^, ^6^. With the development of internal fixation techniques, different patterns using screws with or without the proximate femoral plate have been widely used, and the screws at the proximal end were commonly implanted in a parallel configuration^7^. Current researchers have mixed opinions on this fixation method. Femoral head necrosis, implant disability, and nonunion are common complications of internal fixation in femoral neck fracture postoperatively^8^. According to the current research, the traditional parallel cannulated screws were associated with reduced stability under the directional loading and high complications in clinical cases^9^, ^10^.

Research on the effectiveness of internal fixation involves multiple fields, including anatomy studies, biomechanical studies, and comparative clinical trials. One of the important factors that affect the implant selection and mechanical properties of femoral neck internal fixation is the spatial shape of the femoral neck canal, especially the morphological characteristics of the cross‐section of the femoral neck isthmus, which directly affects both the spatial configuration and type of the cannulated screws^7^. Previous studies have shown that the cross‐section of the femoral neck gradually changed from a circle to an oval shape from the proximal to distal ends^11^, ^12^. Du *et al*. found that the cross‐section of the femoral neck of a gorilla and a modern human gradually changed from a circular shape to an oval shape from the proximal to the distal end^13^. The cross‐sectional shape of the proximal end of the femoral neck was found to be closer to a circle in the modern human, and the transition of the femoral head and neck was relatively smooth. This change was mainly due to the mechanical load generated by the two walking modes. Also, a few researchers assumed that the cross‐sectional shape of the femoral neck may be triangular^14^.

Currently, the diverse configuration of implants is controversial among surgeons and causes subjective deviations and mismatching of implants^11^, ^12^, ^15^. The ideal insertion of screws should provide enough support in multiple directions and minimize the iatrogenic injuries in the femoral neck. Therefore, the morphological evidence about the femoral neck, especially the isthmus, was elementary for the cannulated screws in clinical use. However, current knowledge of the morphological characteristics of the cross‐section of the femoral neck based on visual observation or inference using computer regression analysis lacks corresponding theoretical support. Bonneau *et al*. compared the single‐cylinder method with the two‐angled cylinder method and found that the continuous ellipse method using the 3D reconstruction was more accurate in identifying the morphology of the femoral neck. Moreover, the regression analysis showed that the continuous cross‐section ellipse model might be a better way to describe the structure of the femoral neck^11^, ^12^.

The purpose of this study was to: (i) reconstruct and reslice the cross‐section of the FNI according to inertia axis in the 3D model; (ii) employ the mathematical tools to measure and analyze the geometric features of the femoral neck and FNI; (iii) predict factors associated with the area of the FNI cross‐section using correlation analysis, and calculate equations using regression analysis to guide the implant of screws in osteosynthesis of femoral neck fractures. This study hypothesized that actual measured values of the cross‐section of FNI were in line with the predicted values using the elliptical equation.

## Methods

### *Patients*

After obtaining the approval of the Institutional Review Board (IRB) of the General Hospital of the Chinese People's Liberation Army, computed tomography (CT) data of bilateral hip joint from 213 consecutive patients who underwent surgical treatments for proximal femoral fracture at our hospital from December 2009 to December 2012 were retrospectively reviewed. The patients received a completed CT scan (Somatom Sensation 16, Siemens AG, Erlangen, Germany) of the bilateral hip joint and the slice thickness was 1.2 mm. The exclusion criteria are set as follows: (i) patient with a history of femoral neck fractures or surgery of the hip joint; (ii) patient with a history of congenital deformities, like developmental dysplasia of the hip, coxa vara, and retroversion; (iii) patient with necrosis of femoral head; and (iv) patient with severe hip or knee arthritis. Two hundred patients with bilateral CT images of the hip joint were included in this study. The study population comprised 137 males and 63 females with a mean age of 69.41 ± 9.21 years, ranging from 50–85 years. The group’s demographic data according to gender was listed in Table 1.

**TABLE 1:** Patients' demographics (Mean ± SD)

Demographic	Male (*n* = 137)	Female (*n* = 63)	*P*‐value
Age (years)	69.13 ± 9.53	69.68 ± 8.55	0.475
Height (meters)	1.68 ± 0.06	1.59 ± 0.06	<0.001
Weight (kg)	66.48 ± 8.89	62.32 ± 9.80	<0.001
BMI (kg/m^2^)	23.46 ± 2.74	24.77 ± 3.54	<0.001

### *Reconstruction Method of the Femoral Neck Isthmus*

**Fig. 1:**
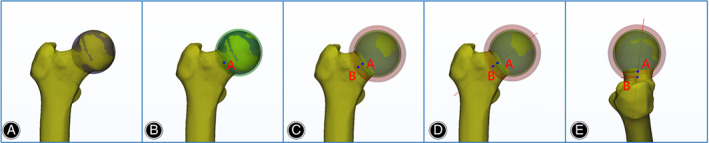
Method for determining the 3D axis of the femoral neck. (A) The first sphere was set to fit the femoral head. (B) The second sphere was formed with the same center as the first sphere with an increase in the radius of 2 mm over that of the first sphere; this sphere was crossed with the femoral neck to obtain a corresponding curve. The curve was fitted to an arc, and the center of the fitted arc was defined as point A. (C) The third sphere was established with the same spherical center, and the radius was increased by 5 mm again; this sphere was also crossed with the femoral neck. The fitted arc was built with the previous method, in which the center was defined as point B. (D,E) The line connecting point A to point B was defined as the three‐dimensional axis of the femoral neck.

In this study, the 3D model of the proximal femur was reconstructed using Mimics 20.0 software (Materialize N.V, Belgium) according to the standard methods^16^, ^17^. The reconstructed model was then imported into 3‐Matic 12.0 software (Materialize N.V, Belgium) in STL format. The femoral head surface was marked using the “Wave Brush Mark” method, then the marked triangles of the femur head were analyzed in the software to create the first sphere^18^. The second sphere was formed with the same center as the first sphere, and with an increase of 2 mm on the radius. The sphere that crossed the femoral neck was marked to obtain a corresponding intersecting line (the “Curve” method). The intersecting line was fitted to an arc. The center of the fitted arc was defined as point A. The third sphere was created with the same spherical center, and the radius was increased by 5 mm, crossing the femoral neck to form a fitted arch, and the center of which was defined as point B. The straight‐line connecting point A and point B was defined as the 3D axis of the femoral neck (Fig. 1). About the method used to determine the femoral isthmus, a continuous vertical section of the femoral neck along its 3D axis with a 1‐mm interval between adjacent sections was made. The cross‐section with the smallest cross‐sectional area was defined as the FNI^19^. The Section method in 3‐Matic software was used to obtain a vertical section of the 3D axis of the femoral neck, and the cross‐sectional area of the FNI was read directly in the software. A plane was created to show the contour of the FNI cross‐section.

### *Parameters of the FNI*

**Fig. 2:**
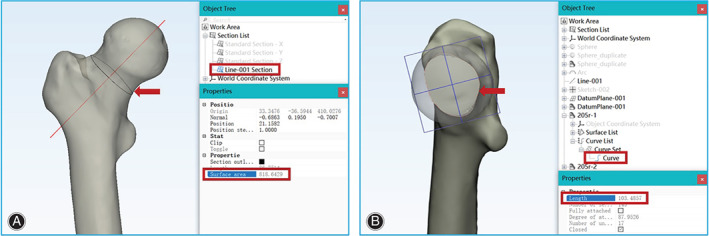
The area and perimeter of the femoral neck cross‐section were read directly by 3‐Matic software. (A) The Section function in the software was used to make a cross‐section along the 3D axis of the femoral neck; then, the area of each section could be read directly. (B) A plane of size 25×25 mm was established through the cross‐section of the FNI. The Curve function in the software was used to make the plane intersect with the femoral neck to generate a curve. The length of the curve directly read by the software was the perimeter of the cross‐section.

The perimeter and area of the FNI cross‐section were automatically calculated in the 3‐Matic and defined as the actual measured value (Fig. 2).

**Fig. 3:**
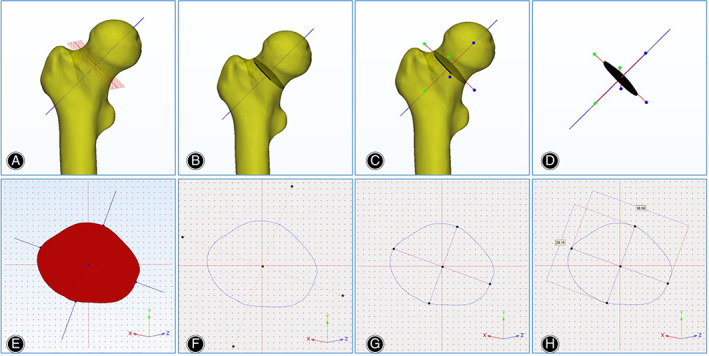
The long and short axes of the cross‐section of the FNI were measured with the “inertia axis” method. (A) The 3D axis and the cross‐section of the isthmus of the femoral neck were built in 3‐Matic software. (B) The cross‐section was extruded to an extruded part with a 0.5‐mm depth. (C, D, E). The inertia axes of the extruded part of the cross‐section were established with the “fit inertia axes” method. (F) The inertia axes were projected in the sketch of the cross‐section. (G, H) The long and short axes of the FNI were acquired and measured.

The “inertia axis” method was used to determine the long and short axes of the FNI cross‐section^20^, ^21^, ^22^. Two perpendicular lines on the cross‐section of the FNI center on the inertial axes were defined as the long axes and short axes. The long diameter was defined as the line connecting the vertices of the upper part and the lower part of the FNI (Fig. 3C). The line perpendicular to the long diameter was defined as the short diameter. The length of the long diameter and short diameter were measured as A and B, and the radius was a and b. The elliptical equations were used to predict the cross‐sectional area, perimeter, and eccentricity of the FNI^23^. The perimeter of the ellipse was calculated according to the formula 2πb + 4(a‐b), the area was calculated according to the π × a × b, and the eccentricity of the ellipse was calculated according to the √[1‐(b/a)^2^].

### *Statistical Analysis*

All data were analyzed using SPSS software (Version 21.0, IL, USA). All measurements had passed the normality test, and then the paired t‐test was used to compare the difference between the calculated area and perimeter and the measured values. Furthermore, the area and perimeter of the FNI were also grouped by sex and sides and compared. Pearson's Correlation Analysis was then used to elucidate the correlation between the morphological parameters of the cross‐section of the femoral neck and the patient's demographics (age, height, weight, and body mass index [BMI]). Finally, the factors that affected the morphological parameters of the cross‐section of the femoral neck were determined by stepwise linear regression analysis.

At least 180 samples were required for the linear regression (*α =* 0.05, 1‐*β* = 0.65) according to the study by Walter and Eliasziw *et al*.^24^ The Intra‐class Correlation Coefficient (ICC) was used to evaluate the reliability of the measurements established in this study. Two senior orthopaedic surgeons (RYZ and YPZ) independently measured the parameters of the 30 random samples twice with an interval of 4 weeks to assess the interobserver reliability. A *P*‐value of less than 0.05 was statistically significant. Finally, measurements of 200 patients were randomly numbered using the digital table method, and data of a senior physician (RYZ) was adopted for this study.

## Results

### *General Results*

The ICC results were excellent, with values from 0.989 to 0.996, which indicated high reliability and reproducibility (Table 2). The *t*‐test showed that all morphological parameters were significantly larger in males than females (Table 3). There was no significant difference in morphological parameters between the left and right sides (Table 4).

**TABLE 2:** Intraobserver and interobserver reliability of the measurements

Indexes	Intraobserver Reliability		Interobserver Reliability	
	ICC	95% *CI*	ICC	95% *CI*
Perimeter (measured value)	0.992	0.987–0.996	0.996	0.990–0.999
Perimeter (predicted value)	0.989	0.979–0.994	0.995	0.989–0.997
Area (measured value)	0.991	0.988–0.996	0.996	0.989–0.999
Area (predicted value)	0.993	0.984–0.995	0.995	0.991–0.999

CI, Confidence Interval; ICC, Intraclass Correlation Coefficient.

**TABLE 3:** Paired‐samples *t*‐test of the area and perimeter (Mean ± SD, mm^2^, mm)

Value	Total (*n* = 400)		Males (*n* = 274)		Females (*n* = 126)		Left (*n* = 200)		Right (*n* = 200)	
	Area	Perimeter	Area	Perimeter	Area	Perimeter	Area	Perimeter	Area	Perimeter
Measured value	732.83 ± 126.74	97.86 ± 8.60	817.89 ± 96.05	103.78 ± 6.00	636.96 ± 80.14	91.20 ± 5.74	730.83 ± 127.66	97.74 ± 8.62	734.83 ± 126.27	97.99 ± 8.62
Predicted value	731.62 ± 128.15	92.84 ± 8.65	817.33 ± 96.97	102.73 ± 6.00	635.03 ± 81.99	90.21 ± 5.96	730.97 ± 129.31	92.81 ± 8.72	732.28 ± 127.45	92.87 ± 8.62
*P*‐value	0.322	<0.001	0.769	<0.001	0.176	<0.001	0.935	<0.001	0.131	<0.001

**TABLE 4:** Differences in the area, perimeter, and eccentricity (Mean ± SD) between sex and sides

Indexes	Males (*n* = 137)		Females (*n* = 63)		*P* ^1^	*P* ^2^
	Left	Right	Left	Right		
Area	815.87 ± 97.70	819.92 ± 95.02	634.99 ± 81.03	638.93 ± 79.84	<0.001	0.715
Perimeter	103.5 ± 6.05	103.98 ± 6.00	91.17 ± 5.92	91.23 ± 5.60	<0.001	0.747
Eccentricity	0.61 ± 0.07	0.60 ± 0.08	0.58 ± 0.07	0.58 ± 0.06	0.003	0.694

*P* ^1^, the *p*‐value for sex; *P* ^2^, the *P*‐value for sides.

### *Comparison of the FNI Area and Perimeter*

The actual measured value of morphological parameters in the FNI cross‐section was in line with the predicted value using the elliptic equations. There was no significant difference in the cross‐sectional area of the FNI between the actual measured values and the predicted values using the formula (732.83 ± 126.74 mm^2^ *vs* 731.62 ± 128.15 mm^2^, *P* = 0.322). The perimeter calculated by the two methods showed a narrow while significant difference (97.86 ± 8.60 mm *vs* 92.84 ± 8.65 mm, *P* < 0.001), the actual measured values were about 5 mm greater than the predicted values (Table 3).

### *Results of Correlation Analysis*

The correlation results between the morphological parameters of the femoral neck cross‐section and the parameters of the demographics were listed as following (Table 5): The patient height had shown a strong correlation with the cross‐sectional area (*r =* 0.703, *P <* 0.01), perimeter (*r =* 0.695, *P <* 0.01), and weak correlation with the elliptical eccentricity of the FNI (*r =* 0.159, *P <* 0.01). In addition, there was a moderate correlation between the patient weight and cross‐sectional area (*r =* 0.390, *P <* 0.01), perimeter (*r =* 0.374, *P <* 0.01). There was also a slightly negative correlation between the cross‐sectional area of the isthmus and the age of patients was observed (*r* = −0.206, *P* < 0.01).

**TABLE 5:** Correlation (*r*‐value) between morphological parameters of the FNI and participant characteristics

Indexes	Age	Height	Weight	BMI
Area (measured value)	−0.206^*^	0.703^*^	0.390^*^	0.008
Perimeter (measured value)	−0.183	0.695^*^	0.374^*^	0.044
Eccentricity (predicted value)	−0.043	0.159^*^	0.005	0.093

* The correlation was significant at the level of 0.01 (two‐tailed).

### *Regression Equation of the FNI Area*

Results of the stepwise linear regression showed that the regression equation of the cross‐sectional area of the FNI was: *Y* = −1083.75 + 1033.86 × HEIGHT + 1.92 × WEIGHT, and *R* ^2^ = 0.489.

## Discussion

In this study, the main finding was there is no significant difference between the actual measured value and the equation‐predicted value on the cross‐sectional area of the FNI. There was a significant difference in the perimeter of FNI between the two methods, the actual measured value was larger than the calculated value, and the difference was narrow (approximately 5 mm). The results indicate that the cross‐section of the femoral neck could be described using the elliptic equations, and has an approximate oval‐like shape mathematically.

Regarding the narrow gap in the perimeter of FNI between the actual value and the formula value, there may be two main factors affecting this result. One is the elliptic equation in the mathematical model is not completely suitable for the FNI due to the systematic bias, and the other is that the inner surface of the femoral neck is not smooth. Generally, the cross‐sectional perimeter of a smooth object was smaller than that of an object with an equal cross‐section but with a rough surface.

The correlation analysis showed a highly positive correlation between the cross‐sectional area or perimeter and height, which is consistent with the effect of height on bone morphology that has been reported in the literature, namely, that height is proportional to the bone development of the femoral neck^25^. In addition, a moderate positive correlation between the cross‐sectional area or perimeter and body weight was observed. This finding was likely because greater weight causes more loading on the bone, and the increase in mechanical stimulation leads to increased size. A slightly negative correlation between the cross‐sectional area of the isthmus and age was observed. The result may be due to the loss of bone ingredients with aging.

Surgical treatments of the femoral neck fractures were not yet perfect for orthopaedic doctors. Minimally invasive internal fixation is the first choice for young and middle‐aged patients. Studies regarding internal fixation treatments for femoral neck fractures have shown that surgeons still prefer using three screw fixations in a parallel pattern. Due to the unique anatomical structure of the femoral neck, the incidence of postoperative complications (fracture nonunion, femoral head necrosis, and deformity healing) can reach up to 40%^2^, ^26^. And the optimal configuration of the screws is controversial^15^, ^27^, ^28^. One of the factors that greatly affect the configuration is the 3D shape of the femoral neck, especially the shape of the cross‐section of the FNI. Biomechanical and clinical efficacy observations have shown that an inverted equilateral triangle configuration with three screws is superior to other configurations^29^, ^30^. And the classic configuration is designed consistently with an assumption of a circle‐like shape of the cross‐section of the femoral neck.

According to the previous studies and our research finding, the morphology of the cross‐section of the femoral neck is more like an ellipsis. Furthermore, some researchers have reported that the femoral neck has a specific torsion angle^15^, ^31^. Therefore, we supposed that the morphology of the cross‐section of the femoral neck may appear oval‐like and tilted. In summary, to achieve the maximum pullout strength or the maximum mechanical properties under the premise of safe placement of three screws, the spatial distribution of the three screws should be as close as possible to the contour of the FNI. Despite the torsion angle of the femoral neck, the morphological characteristics including the eccentricity, area, and perimeter of the FNI might be the considerable factors that determine the spatial configuration of the three screws. Therefore, when three screw fixations in a parallel pattern were used for the treatment of femoral neck fractures, the spatial configuration of the three screws may not be a standard inverted isosceles triangle, it may be an oblique triangle or an isosceles triangle with a specific incline angle^15^, ^28^. Moreover, for the steel plates that are currently used to treat femoral neck fractures, the spatial configuration of the three screws at the proximal end of the plate should be adapted to the major morphological features of the cross‐section of the femoral neck to achieve the maximum fixation strength and fewer complications of screw cutout^27^, ^32^. Due to variation in the anatomical shape of the femoral neck, the cannulated screw configuration and steel plate size should be individually selected according to the shape of the femoral neck on the healthy side before surgery. Precise preoperative planning aided by computer stimulation or 3D‐print may be a trend in the treatment of femoral neck fractures in the future^12^, ^33^.

### *Limitations*

This study has several limitations. One is the use of the outer boundary of the femoral neck cortex to analyze the morphology of the cross‐section of the femoral neck, which does not completely reflect the morphology of the medullary cavity of the femoral neck out of the cortex thickness. This might explain the difference between the actual measured value and software value. The other is that we measured the characteristics of the cross‐section of the FNI but did not study the morphological changes in the femoral neck from the proximal to the distal end. Researchers need a more continuous analysis of the femoral neck based on approximate anatomical landmarks. At last, the *R* ^2^ value was 0.489 in the regression equation of the cross‐sectional area of the FNI. It is worthy mentioning that the more the value approached +1 or − 1, the more it fits the model. Therefore, further investigations are needed to strengthen the robustness of the equation.

## Conclusions

This study supported the notion that the cross‐section shape of the FNI appears to be oval‐like in the 3D model, separated according to the inertia axis. The size of the FNI cross‐section was correlated with height and weight. The findings of this study proposed an anatomical basis for further study of the spatial configuration of cannulated screws in the treatment of femoral neck fractures.

### *Author contributions statement*

LCZ and PFT conceived the study. RYZ and YPZ wrote the manuscript. RYZ, YPZ, and XYS performed the study. RYZ, YPZ, XYS, JTL, and ZJX contributed to the data collection and interpretation of the results. All authors read and approved the final manuscript and consented to publish this manuscript.
